# Research on exercise fatigue estimation method of Pilates rehabilitation based on ECG and sEMG feature fusion

**DOI:** 10.1186/s12911-022-01808-7

**Published:** 2022-03-18

**Authors:** Dujuan Li, Caixia Chen

**Affiliations:** 1 North Sichuan Medical College, Nanchong, 631000 China; 2 China West Normal University, Nanchong, 631000 China

**Keywords:** Exercise fatigue, Surface EMG signal, Electrocardiogram signal, Feature fusion, Particle swarm optimization algorithm

## Abstract

**Purpose:**

Surface electromyography (sEMG) is vulnerable to environmental interference, low recognition rate and poor stability. Electrocardiogram (ECG) signals with rich information were introduced into sEMG to improve the recognition rate of fatigue assessment in the process of rehabilitation.

**Methods:**

Twenty subjects performed 150 min of Pilates rehabilitation exercise. Twenty subjects performed 150 min of Pilates rehabilitation exercise. ECG and sEMG signals were collected at the same time. Aftering necessary preprocessing, the classification model of improved particle swarm optimization support vector machine base on sEMG and ECG data fusion was established to identify three different fatigue states (Relaxed, Transition, Tired). The model effects of different classification algorithms (BPNN, KNN, LDA) and different fused data types were compared.

**Results:**

IPSO-SVM had obvious advantages in the classification effect of sEMG and ECG signals, the average recognition rate was 87.83%. The recognition rates of sEMG and ECG fusion feature classification models were 94.25%, 92.25%, 94.25%. The recognition accuracy and model performance was significantly improved.

**Conclusion:**

The sEMG and ECG signal after feature fusion form a complementary mechanism. At the same time, IPOS-SVM can accurately detect the fatigue state in the process of Pilates rehabilitation. On the same model, the recognition effect of fusion of sEMG and ECG(Relaxed: 98.75%, Transition:92.25%, Tired:94.25%) is better than that of only using sEMG signal or ECGsignal. This study establishes technical support for establishing relevant man–machine devices and improving the safety of Pilates rehabilitation.

## Introduction

Pilates is a combination of strength, flexibility, and balance exercises. It focuses on lumbopelvic stabilization, with the activation of the deep muscles of the trunk, and seeks a complete connection of body and mind [1]. The core muscles provide balance and strength for Pilates, so exercise plays an important role in women’s postpartum recovery [2], prevention of low back pain and rehabilitation [3], spinal health correction [1, 4]. In the process of Pilates exercise, program-controlled human–computer interaction equipment, such as medical rehabilitation robot and exoskeleton robot, is to help patients complete the set movement. However, the muscle fatigue information is rarely used as an influencing factor to adjust the rehabilitation process. That not only has a great impact on the recognition rate of patients’ motor intention but also tends to cause secondary injuries and reduce the rehabilitation effect.

Common detection techniques for muscle fatigue include surface electromyography (sEMG) [5], muscle sound signal [6], muscle oxygen saturation [7], etc. When muscle activity or biochemical characteristics change, these signals change accordingly. Muscle sound signal and muscle oxygen saturation are expensive and difficult to obtain. So sEMG has many achievements in the field of online monitoring and processing of muscle fatigue. Gongfa Li et al. [4, 8–10] studied the prosthetic hand grasps the object base on the forearm electromyography signal, and the result of surface EMG signal decoding is applied to the controller, which can improve the fluency of artificial hand control. Choi Chang et al. [5] developed a computer interface base on sEMG and virtual reality, which can be applied to spinal cord injury patients. They can control the cursor movement by adjusting the level of muscle contraction. Shahmoradi et al. [11] collected the sEMG and Maximum Voluntary Contraction (MVC) data in the rehabilitation process as inputs of the fatigue state recognition model. The hidden Markov model and artificial neural network were studied for fatigue classification of sEMG. The results show the HMM has a better recognition effect with an accuracy of 95.3%. Because fatigue is a complex phenomenon with characteristics of weakness, randomness and low frequency. When sEMG evaluates muscle fatigue under exercise, it is often affected by sweat, environment, heartbeat and so on, the sEMG classification method alone is not stable. Many scholars combine sEMG with other monitoring methods, such as electroencephalogram (EEG) and electrocardiogram (ECG) [12–15].

Compare with EEG, Electrocardiogram (ECG) is one of the most commonly used non-invasive diagnostic tools for recording the physiological activities of the heart over some time. The ECG data [16] contains much information about the human motor function and is widely used in muscle state research and emotion estimation and so on. SEMG signals often have ECG characteristic signals. In the previous sEMG body state prediction, it is often necessary to eliminate the characteristic signals of ECG. But there is no accurate standard for this. It brings many difficulties to the evaluation research.

Considering the muscle fatigue characteristics of the sEMG and ECG, it is of great significance to establish the fatigue state recognition model of the Pilates rehabilitation process by the fusion sEMG and ECG [17]. Data fusion will produce high-dimensional data, so it is necessary to extract fatigue related features from the fused data of sEMG and ECG, and improve the existing particle swarm classifier according to the data features.

The fatigue degree of the subjects after the Pilates rehabilitation was divided into 6–20 score ranges by the scale for Rating of Perceived Exertion (RPE scale) [18]. The segments from 6 to 10, 13–14, 17–18 scores in the table were identified as Relaxed, Transition, Tired. The ECG and sEMG signal at the tibialis anterior muscle and semitendinosus muscle of the lower limbs were collected while doing the established actions of Pilates. A series of preprocessing was performed to extract the feature variables, which were used as the improved particle swarm optimization support-vector. The input volume of the machine classifier, which achieves iterative optimization of the fusion of complex signals, high-dimensional features, and accurate identification of the three motion states. The advantages and disadvantages of this method were analyzed by the recognition effect.

## Methods

### Data collection

In this section, the data collection will be described, and analysis methods will be explained in detail. The data has been obtained from 20 physical health subjects (22–26 years old; 8 males, 12 females).

The experiments were conducted using Trigno Wireless Systems and Smart Sensors. The Trigno Wireless EMG system is a very popular device with simple and reliable performance. Each EMG sensor has a built-in triaxial accelerometer. Its signal can be transmitted in 40 m and can be detected continuously for 8 h. The system can transmit the data stream to EMGworks 4. Acquisition and analysis software for generating 16 EMG sensors (37 mm × 26 mm × 15 mm) and 48 accelerometer analog channels for integration with motion capture and other third-party data acquisition systems. The complete trigger function further expands the possibility of integration with other measurement technologies. The sensor used can respond immediately to the interference detected on the skin surface.

The North Sichuan Medical College conducted this research project by the ethical code of the World Medical Association. It was also approved by the Ethics Committee of the North Sichuan Medical College (No. 2020ER(R)017). This paper took 20 physical examiners as the research object. Selection criteria: full-time students majoring in Physical Education; aerobics as the main special sport; the subjects were in good physical condition, had no obvious disease, and had no damage to the lower limb muscles and knees. The subjects had an average height of 162.3 ± 1.2 cm, an average body weight of 63.5 ± 2.3 kg, and an age of 21.2 ± 1.1 years. Before the experiment, the experimental process was explained to the subjects. All subjects voluntarily participated in the experiment and signed written informed consent. The test time was September 11–25, 2021.

**Fig. 1:**
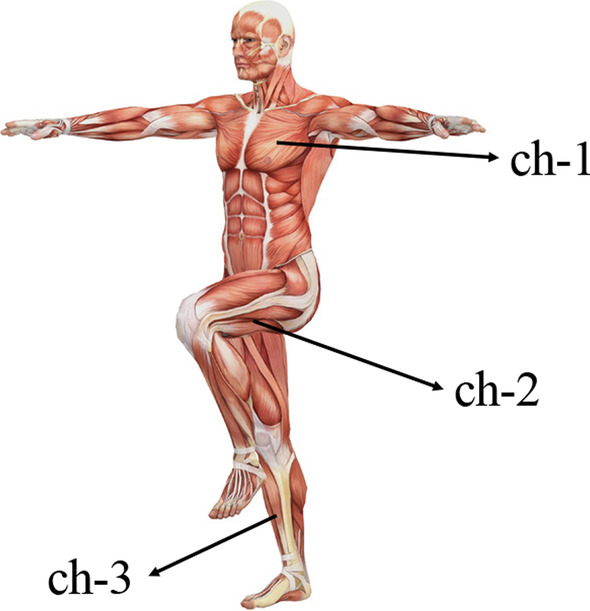
Sensor placement

According to the physiological structure of the human body, the ECG signal and the sEMG signal at the anterior tibialis muscle and semitendinosus muscle were collected synchronously. The sampling frequency was 2 kHz. The sensor position is shown in Fig. 1. Ch-1 is the ECG sensor, ch-2 and ch-3 are the sEMG sensors at the semitendinosus muscle of the right leg and the anterior tibialis muscle of the left leg, respectively.

The subjects were divided into two groups of 10. The prescribed Pilates movement training was carried out according to the plan. It was stipulated that 15 min was a training cycle. Ten subjects completed one cycle of training in turn as a group of experiments. The duration of each experiment was 150 min, and a total of 40 groups of experiments were carried out. The first group finished the test, the second group continued the test, and the first group rested. In each training cycle, the subjects return a calm standing state every 30 s according to the RPE scale [18], report their feelings of fatigue state, and mark the fatigue state value at this time (relaxed: − 1, transition: 0, tired: 1).

**Fig. 2:**
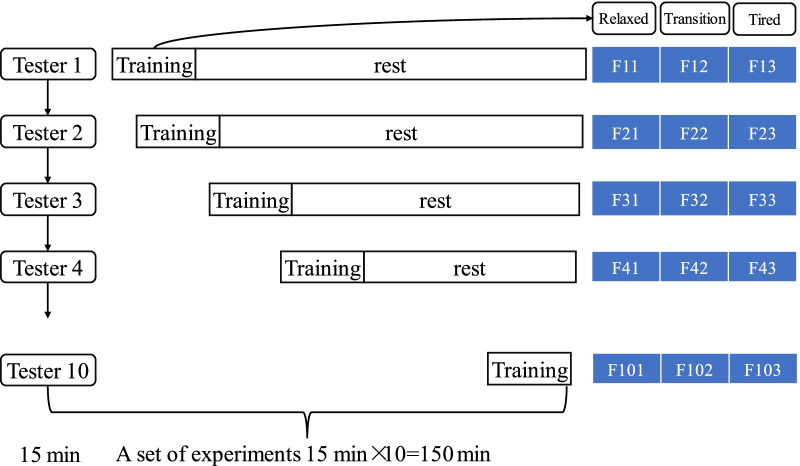
A set of the experimental processes and the feature data obtained

The sEMG and ECG signal in three states were marked and saved, and the corresponding training time was recorded. 30 groups of sEMG and ECG signal were obtained in each group experiment. According to the corresponding fatigue value, each data was divided into three states, with a total of 90 sEMG and ECG data. After the experiment, 3600 sEMG and ECG data were collected respectively. One set of experimental processes and obtained characteristic data are shown in Fig. 2. The signal acquisition and analysis process of all subjects were the same. Here, take one of them as an example.

### Signal preprocessing and fatigue feature extraction

**Fig. 3:**
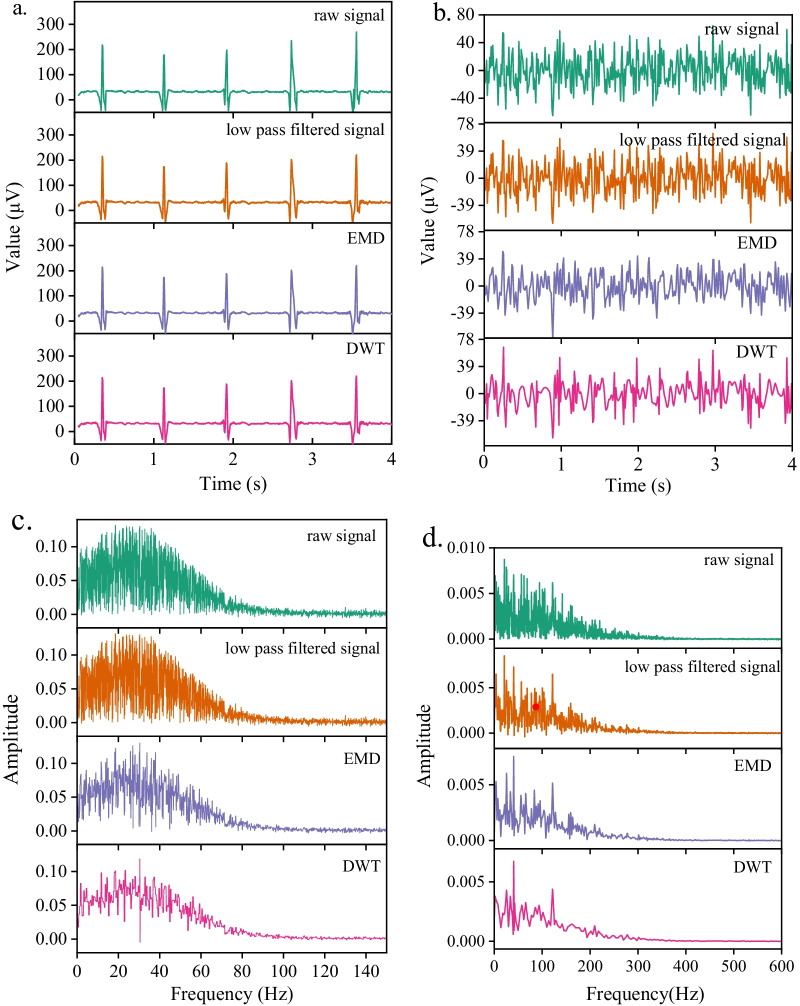
Example of signal preprocessing process. **a** Time-domain signal of ECG, **b** time-domain signal of sEMG, **c** frequency-domain signal of ECG and d. frequency-domain signal of sEMG

The original sEMG and ECG signal contains noise interference, which needs to be preprocessed. Firstly, the original ECG and sEMG signals were filtered by 0–100 Hz and 0–500 Hz low-pass filters to remove high-frequency interference. Secondly, 49.5–50 Hz adaptive notch filters were used to filter the power frequency and harmonic interference in the signal. Finally, empirical mode decomposition(EMD) and discrete wavelet transform(DWT) domains were used to reduce the noise [19]. which reduce the noise from the initial IMFs instead of discarding them completely thus yielding a relatively cleaner ECG signal [20]. MATLAB 2021a was used to analyze and process the collected data. The time-domain and frequency-domain data processing of ECG and sEMG are shown in Fig. 3.

The calculation method of specific parameters with an asterisk in the Table 1 is as follows [22]:

1 $$ECG_{{{\text{mean}}}} = \frac{1}{M}\sum\limits_{i = 1}^{M} {\left( {RR_{i} } \right)}$$

**Table 1:** Fatigue-related physiological features of ECG and sEMG [21]

Features	Features description
ECG_mean_	*Mean of the ECG interval sequence
ECG_LF_	Interphase sequence low-frequency band power (0.04–0.15 Hz)
ECG_LF/HF_	ECG interphase sequence with low/high band power ratio
sEMG_R-IEMG_	sEMG of the musculus semitendinosus in the right leg
sEMG_R-RMS_	*The mean square root of sEMG_R-IEMG_
sEMG_R-MPF_	*The mean power frequency of sEMG_R-IEMG_
sEMG_R-MF_	*The median frequency of sEMG_R-IEMG_
sEMG_L-IEMG_	EMG of tibialis anterior in the left leg
sEMG_L-RMS_	The mean square root of sEMG_L-IEMG_
sEMG_L-MPF_	The mean power frequency of sEMG_L-IEMG_
sEMG_L-MF_	The median frequency of sEMG_L-IEMG_

In the above relations, *RR*_*i*_ is the duration of ECG interval; *M* is the total number of periods.

2 $$sEMG_{{R{ - }IEMG}} = \int_{t}^{t + T} | x(t)|{\text{d}}t = \sum\limits_{{k = N_{1} }}^{{N_{2} }} | x(k)| \times \frac{1}{{F_{s} }}$$

3 $$sEMG_{{R{ - }RMS}} = \sqrt {\frac{1}{N}\int_{t}^{t + T} | x(t)|{\text{d}}t} = \frac{1}{N}\sum\limits_{{k = N_{1} }}^{{N_{2} }} x (k) \times \frac{1}{{F_{{\text{S}}} }}$$

4 $$sEMG_{R - MPF} = \int_{{f_{mid} }}^{ + \infty } f P(f)df/\int_{0}^{ + \infty } P (f)df$$

5 $$sEMG_{{R{ - }MF}} = \frac{1}{2}\int_{0}^{ + \infty } P (f)df$$

where *x*(*t*) is the amplitude of the sEMG signal, *x*(*k*) is the amplitude of sEMG signal after discretization, *F*_s_ is sampling frequency. *N*, *N*_1_, and *N*_2_ are the length of sEMG signal, *P*(*f*) is the power spectral density function [23].

### Improved particle swarm optimization-support vector machine (IPSO-SVM) classifier

Traditional feature fusion with constant weights attempts to merge multiple feature vectors into a vector, which performs poorly in muscle fatigue recognition since feature weights cannot change with the testing object [24]. In this study, the multi-class support vector machines (SVMs) are constructed by feature fusion coefficients of particle swarm optimization (PSO) and one-vs-one (OVO) methods to improve the state classifier. The fusion coefficient based on PSO can well represent weight coefficients and trust degrees of weight coefficients, and learn the fusion features via multi-class SVM to achieve state classification; accordingly, the fitness function can be established based on state recognition rate to perform adaptive iterative optimization on the fusion coefficient, finally achieving effective fusion of fatigue characteristics and accurate state classification. The detailed process of fatigue estimation based on improved PSO-SVM (IPSO-SVM) classifier is as follows:

#### Constructing the fused feature vectors

$f_{i} = \left[ {f_{i1} ,f_{i2} , \ldots ,f_{ia} } \right]$, $e_{i} = \left[ {e_{i1} ,e_{i2} , \ldots ,e_{ib} } \right]$, $i = 1,2, \ldots ,n$ are defined as the feature vectors of ECG and sEMG, where *a,b* are the vector dimension and *n* is the number of samples. $d = \left[ {d_{1} ,d_{2} , \ldots ,d_{a + b} } \right]$ is defined as the fusion coefficient vector, the fused feature vector of ECG and sEMG can be denoted as $x_{i} = \left[ {d_{1} f_{i1} , \ldots ,d_{a} f_{ia} ,d_{a + 1} e_{i1} , \ldots ,d_{a + b} e_{ib} } \right]$, $i = 1,2, \ldots ,n$. Using the fused feature matrix $X = \left[ {x_{1} ,x_{2} , \ldots ,x_{n} } \right]^{{\text{T}}}$ composed of fused coefficient vector *d*, *X* can be divided into the training set *X*_*p*_ and the test set *X*_T_. *X*_*p*_ is used for training the classifier and *X*_T_ is used for validating the classification performance.

#### Constructing multi-class SVM fatigue state classifier

SVM, as a kind of machine learning method based on statistics and the principle of structural risk minimization, performs excellently in addressing nonlinear recognition problems with a small set of samples [25]. Fatigue estimation based on ECG and sEMG can be regarded as a type of linear inseparable multi-class problem, which is exactly the field of expertise of the one-to-one method (OVO) [26]. On the classification of class 3 or more, 2 classes are selected and then merged for classification. In this study, OVO was used for constructing 3 binary SVMs to achieve the effective classification of 3 states.

It is assumed that the training set *X*_p_ contains *m* samples, $X_{P} = \left[ {x_{1} ,x_{2} , \ldots ,x_{m} } \right]^{{\text{T}}}$, $Y_{P} = \left[ {y_{1} ,y_{2} , \ldots ,y_{m} } \right]^{{\text{T}}}$, $y_{i} \in \{ - 1,0,1\}$. *y*_*i*_ can be classified into the following 3 states—relaxed state, transition state, and tired state, with the values of − 1, 0 and 1, respectively. SVM attempts to seek an optimal classification function so that the distance of the function on the hyperplane and the support vector reaches the maximum. The kernel function *φ*(*x*) is used for mapping the sample set to high-dimensional space while satisfying the Mercer condition. The selection of *φ*(*x*) can directly determine the classification performance. Owing to favorable performance and application range, radial basis function is selected as the kernel function of SVM in this study,

6 $$\varphi \left( {x,x_{i} } \right) = \exp \left( { - \left\| {x - x_{i} } \right\|^{{\frac{2}{{\sigma^{2} }}}} } \right)$$

In the case of positive definite *φ*(*x*,*x*_*i*_), the problem of seeking optimal hyperplane can be converted into the following convex quadratic programming problem:

7 $$\min_{\omega ,b,\xi } \frac{1}{2}\left\| \eta \right\|^{2} + C\sum\limits_{i = 1}^{N} {\xi_{i} } \, s.t \, \left\{ {\begin{array}{*{20}l} {y_{i} \left( {\omega \cdot \varphi \left( {x_{i} } \right) + b} \right) \geqslant 1 - \xi_{i} } \hfill \\ {\xi_{i} \geqslant 0 \cdot i = 1,2, \ldots ,N} \hfill \\ \end{array} } \right.$$

where C and $\xi_{i}$ are penalty factor and slack variable, respectively. By introducing the Language coefficient *a*, the convex quadratic programming problem can be converted into the dual problem according to Eq. (7), and the optimal solution α*,η*, b*can thus be obtained by solving the dual problem:

8 $$b^{*} = y_{i} - \sum\limits_{i = 1}^{l} {y_{i} } \alpha^{*} \varphi \left( {x_{i} ,x_{j} } \right)$$

Finally, the SVM classification function based on radial basis function can be expressed as:

9 $$f(x) = {\text{sgn}} \left( {\sum\limits_{i = 1}^{N} {\alpha_{i}^{*} } y_{i} \varphi \left( {x,x_{i} } \right) + b^{*} } \right)$$

where sgn is Step Function, The classifier can thus be constructed.

#### ECG-sEMG feature fusion based on IPSO-SVM

The detailed fusion process of ECG and sEMG signals was described as below.

- Initialization of particle swarms. In this study, the random fusion coefficient matrix $D = \left[ {d_{1} ,d_{2} , \ldots ,d_{q} } \right]^{{\text{T}}}$ is defined as the initial particle swarm, in which $d_{j} = \left[ {d_{j1} ,d_{j2} , \ldots ,d_{ja + b} } \right]$ denotes the fusion coefficient vector, $\sum\nolimits_{k = 1}^{a + b} {d_{jk} } = a + b$, *j* = 1, 2, …, *q.* The maximum number of initialization iterations, *q* denotes the size of particle swarm, *c*_1_ and *c*_2_ learning factors, and *ω* denotes the inertia weight.
- Training of SVM network and calculation of the particle fitness degree. The characteristic samples are fused with the corresponding fusion coefficients of particles to obtain the feature fusion matrix $X = \left[ {X_{{\text{p}}} X_{{\text{T}}} } \right]$, in which is used for network training to obtain the classification function *. The particle fitness degree can thus be obtained by testing *X*_T_ with *f*(*x*).
- Update of particle swarm (optimization of fusion coefficient matrix *D*). For the fitness degree *h*(*d*) of each group of particles after the above step b, the optimal fitness degrees of both individual particle and population are calculated according to Eq. (10), while both velocity *v*_*i*+1_ and position *x*_*i*+1_ of each particle to generate a new population, in which rand() denotes the random number within a range of [0, 1].
  10 $$h_{p} = \max (h({\text{d}})),\quad h_{g} = \max \left( {h_{p} } \right)$$
  11 $$\begin{aligned} v_{i + 1} &= \omega \times v_{i} + c_{1} \times {\text{rand}} () \times \left( {h_{p} (i) - x_{i} } \right)\\ &\quad+ c_{2} \times {\text{rand}} () \times \left( {h_{g} (i) - x_{i} } \right) \end{aligned}$$
  12 $$x_{i + 1} = x_{i} + v_{i + 1}$$
- Step b and Step c are repeated until reaching the optimal fitness degree (*h*_g_ ≥ *h*_*e*_, also referred to as the expected fitness), where *D* denotes the optimal fusion coefficient matrix.

#### Fatigue estimation based on optimal fusion coefficient feature fusion

**Fig. 4:**
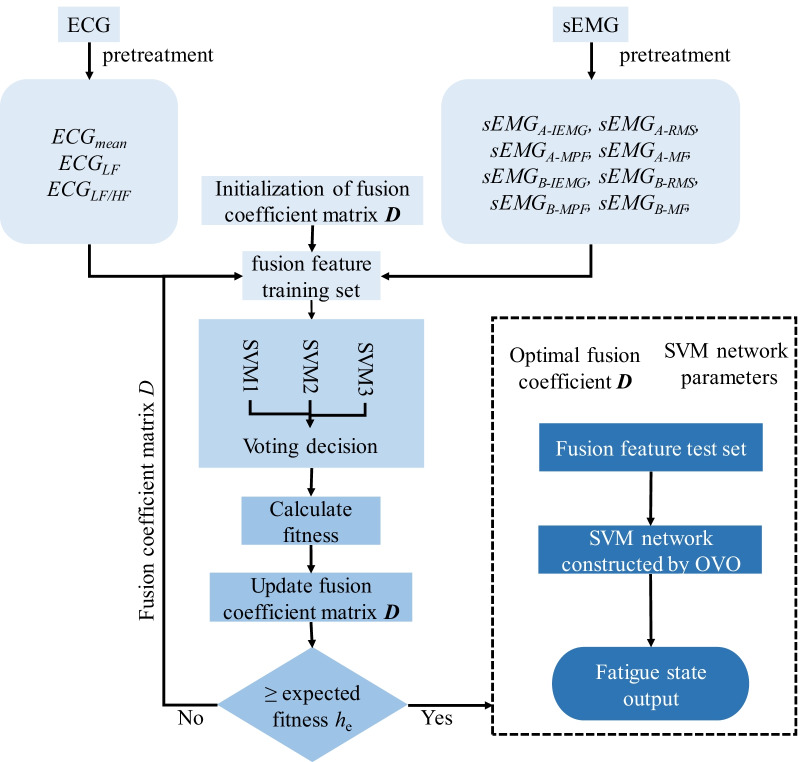
Feature fusion and fatigue estimation process

Using the optimal fusion coefficients, the feature vectors of unknown states are constructed and input to the well-trained SVM network for recognition to achieve accurate classification of fatigue states. The feature fusion and fatigue estimation process was shown in Fig. 4.

In order to better evaluate the performance of the fatigue assessment method designed in this paper, the recognition rate is used as the evaluation index, and the expression is as follows:

13 $$\begin{aligned}{\text{recognition rate}} &= \frac{{\text{number of samples correctly identified}}}{{\text{total number of test samples}}} \\ &\quad\times 100\% \end{aligned}$$

## Results

### Analysis of ECG and EMG physiological features under different fatigue states

**Fig. 5:**
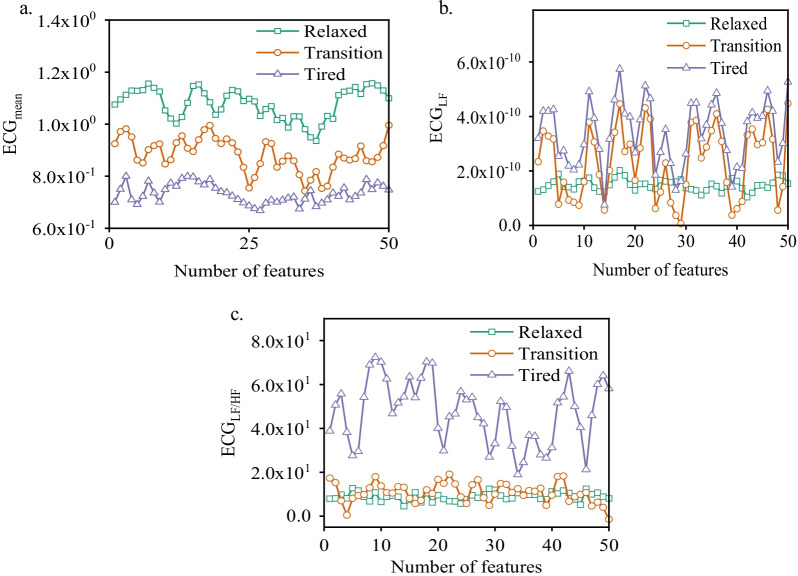
ECG signal features in different fatigue states, **a** ECG_mean_, **b** ECG_LF_, **c** ECG_LF/HF_

**Fig. 6:**
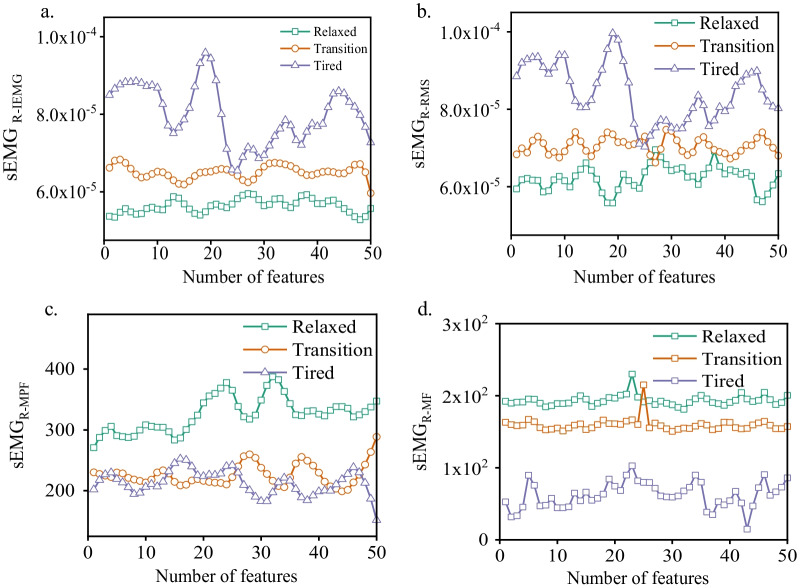
sEMG signal features of the right tibialis anterior muscle under different fatigue states, **a** sEMG_R-IEMG_, **b** sEMG_R-RMS_, **c** sEMG_R-MPF_, **d** sEMG_R-MF_

**Fig. 7:**
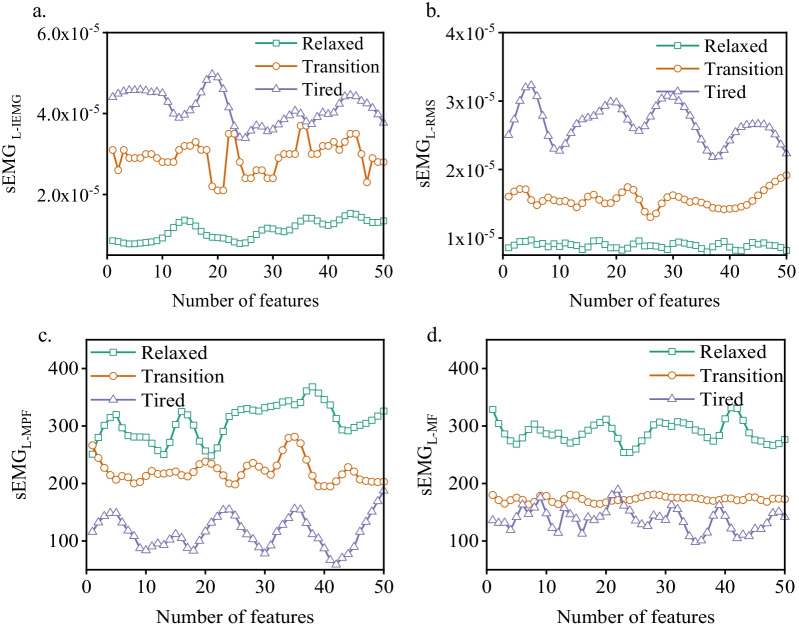
sEMG signal features of the left anterior tibialis muscle under different fatigue states, a. sEMGL-IEMG, b. sEMGL-RMS, c. sEMG_L-MPF_, d.sEMG_L-MF_

Figure 5 shows the ECG signal characteristics of different subjects under different fatigue states. Relaxed and tired states can be easily separated based on ECG signal, but the signal characteristics under transition state overlap with those of the other two states. The characteristics in frequency-domain were particularly intensive than those in the time-domain. Figure 6 shows the sEMG features of the tibialis anterior muscle and semitendinosus of the subjects under different fatigue states. Figure 7 shows sEMG signal features of the left anterior tibialis muscle under different fatigue statesThe sEMG values of muscle integration in the time-domain and root-mean-square (RMS) values show an obvious difference, mean characteristic power frequency and median frequency in frequency-domain overall show obvious tendency; however, the transition state shows a certain overlapping error with the other two states. Both time–frequency characteristics of ECG and sEMG signals in the tired states show obvious fluctuations than those in the other states. Accordingly, the characteristics of ECG and sEMG signals are complementary to some degree. The combination of two types of signals can strengthen the recognition performance of the classifier; however, interference also exists. The characteristic confidence degree, i.e., the fusion coefficient, should be judged and optimized.

### Analysis of fusion coefficient optimization process

**Fig. 8:**
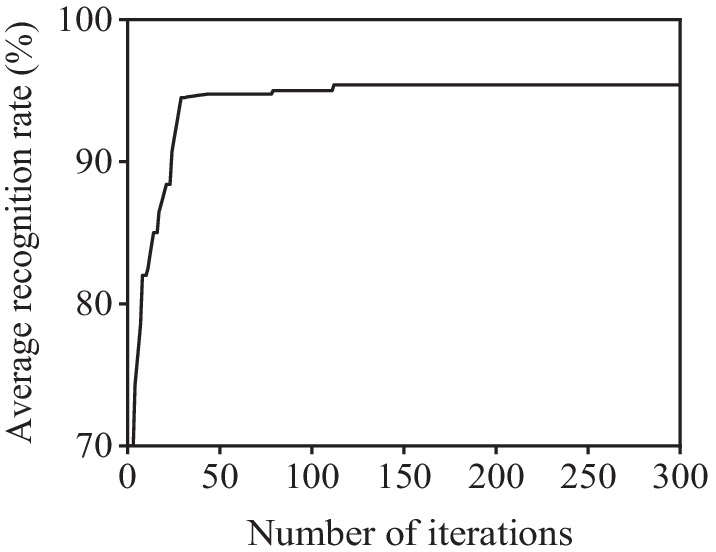
Convergence process of particle swarm optimal fitness

The fusion coefficient is the key to establishing the optimal feature vector and enhancing the fatigue recognition rate. To prevent from falling into local optimum of particle fitness degree, the related parameters in PSO including the population size *q* = 2000, the learning factor *c*_1_ = 0.5 *c*_2_ = 0.5, the inertial weight *ω* = 0.8 and the expected fitness degree can be set as 95%, respectively. 1200 groups of data sets (400 groups for each state) are selected from the collected data for pre-processing and feature extraction; next, the established IPSO-SVM classifier is trained and tested via Monte Carlo cross-validation (MCCV). Figure 8 shows the convergence process of the fitness degree of particle swarm. It can be found that the convergence rate is great for the population with a size of 2000 after 120 iterations.

### Analysis of fatigue recognition results of using different methods

**Fig. 9:**
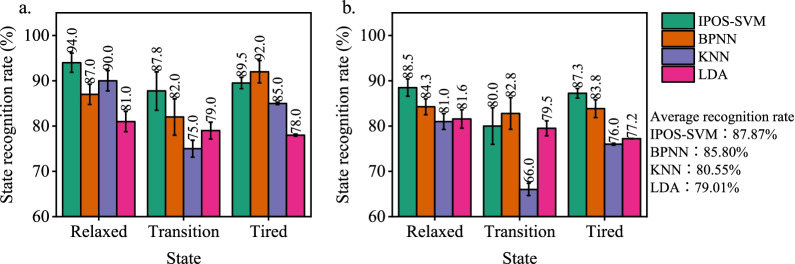
Comparison of recognition results of different classification methods

Some commonly-used classification methods for physiological signals including IPOS-SVM, BP neural network (BPNN), K-nearest neighbor (KNN), and linear discriminant analysis (LDA) were performed on sEMG and ECG signals for training and testing, as the results are shown in Fig. 9 (a. sEMG and b. ECG). It can be found that the IPOS-SVM algorithm showed obvious advantages in the classification of sEMG and ECG signals, with a mean recognition rate of 87.83%; BPNN, as a hotspot in current classification algorithms, was lower than IPOS-SVEM in mean recognition, with a mean recognition rate of 85.80%; KNN was close to LDA in classification performance, with a mean recognition rate of 80.55% and 79.01%. Overall, sEMG showed a favorable fatigue classification performance than ECG, since sEMG data contained more fatigue state characteristics. ECG was poor in the recognition of transition state, which was consistent with previous research results. Through comparison, IPOS-SVM performed well in fatigue state classification; however, the state recognition rate was still quite low (only 87.83%).

**Fig. 10:**
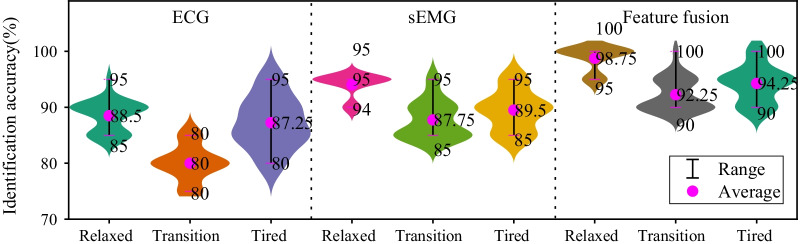
Identification accuracy results based on ECG, sEMG and feature fusion

Aiming at exploring the enhancement of classification performance via data fusion, the classification models are constructed on sEMG signal, ECG signal and the combination of two signals based on IPOS-SVM, as the results are shown in Fig. 10. A violin plot is a boxplot with a rotated kernel density plot on each side. In Fig. 10, it includes a purple point for the average of the data. Overlaid on this box plot is a kernel density estimation.

The recognition rates of relaxed, transition and tired states only with sEMG signal were 94.00%, 87.75% and 89.5%, respectively, while the recognition rates of relaxed, transition and tired states only with ECG signal were 88.5%, 80.00% and 87.25%, respectively. By contrast, sEMG was more sensitive to fatigue state and rich in fatigue state information. ECG showed a poorly recognition rate of the transition state (only 80%). After feature fusion of sEMG and ECG, the recognition rates of the relaxed state, the transition state and the tired state could be remarkably enhanced to 94.25%, 92.25% and 94.25%, respectively. The recognition rate of transition rate exceeded 90%, which can be explained by the following two reasons. Firstly, ECG features can contribute to recognizing interference variables and play the role of correction. Secondly, IPOS-SVM can perform distribution based on the trust degrees of high-dimensional characteristics after multiple iterative computations, which can assign appropriate weights in different cases.

## Discussion

The fatigue produced in Pilates is a complex phenomenon in rehabilitation exercises. How to enhance the accuracy of fatigue estimation based on feature fusion of multi-source physiological signals appears as an effective mean. However, due to the lack of uniform research paradigm and standards, many studies have been stuck on laboratory or special application scenarios. Both sEMG and ECG are nondestructive body monitoring signals abundant in physical information. Establishing the classification model or quantitative model based on the combination of sEMG and ECG shows huge potential. This study starts from the perspective of fatigue in Pilates and proposes a lower limb fatigue estimation method based on sEMG and ECG to achieve the classification of 3 states (relaxed, transition, and tired states) in the lower limb rehabilitation process. The classification model by integrating ECG and sEMG fatigue features into fatigue states is established with IPOS-SVM. Results also confirm better classification performances of IPSO-SVM than BPNN, KNN and LDA, i.e., the proposed IPSO-SWM is appropriate for the classification of fatigue states based on sEMG and ECG signals. IPSO-SVM classification model based on surface electromyography and ECG fusion features had good processing ability for high-dimensional feature information, and can well identify 3 fatigue states with the recognition rates of 94.25%, 92.25% and 94.25%, respectively. The mean recognition rate was 93.58%. Compared with the study report of Shangbin Li [27] (Reconition rate: 83.15–93.62%), the recognition rate of the model in this paper is improved. By comparison with the results based on pure sEMG and pure ECG signals, the model based on feature fusion shows better recognition precision and performance. Conclusively, sEMG and ECG signals can be combined for feature fusion to achieve accurate fatigue detection during the Pilates rehabilitation process, which can lay a solid foundation for further constructing the related man–machine device and enhancing the safety of Pilates rehabilitation.

It must be admitted that there are deficiencies in this study. IPSO-SVM in the paper pays more attention to enhancing the recognition rates of different fatigue states. Compared with single detection means, the operability of operators and the complexity should be further optimized. Meanwhile, this study focused on the recognition of 3 discrete states during the rehabilitation process. In future studies, our team will attempt to explore the mapping relations between continuous fatigue states and ECG/sEMG signals to establish a more accurate quantitative model of muscle state.

## Data Availability

The datasets generated and analysed during the current study are not publicly available due the need for other teachers in my team to carry out other research but are available from the corresponding author on reasonable request.
